# B cell infiltration is highly associated with prognosis and an immune-infiltrated tumor microenvironment in neuroblastoma

**DOI:** 10.20517/2394-4722.2021.72

**Published:** 2021-06-06

**Authors:** Evelien Schaafsma, Chongming Jiang, Chao Cheng

**Affiliations:** 1 Department of Molecular and Systems Biology, Dartmouth College, Hanover, NH 03755, USA.; 2 Department of Biomedical Data Science, Geisel School of Medicine at Dartmouth, Lebanon, NH 03756, USA.; 3 Department of Medicine, Baylor College of Medicine, Houston, TX 77030, USA.; 4 Dan L Duncan Comprehensive Cancer Center, Baylor College of Medicine, Houston, TX 77030, USA.; 5 The Institute for Clinical and Translational Research, Baylor College of Medicine, Houston TX, 77030, USA.

**Keywords:** Neuroblastoma, immune cell infiltration, prognosis, MYCN amplification, B cells

## Abstract

**Aim:**

Neuroblastoma is the most common extracranial solid tumor in children. Recent advances in immunotherapy Approaches, including in neuroblastoma, have shown the important role of the immune system in mounting an effective anti-tumor response. In this study, we aimed to provide a comprehensive investigation of immune cell infiltration in neuroblastoma utilizing a large number of gene expression datasets.

**Methods:**

We inferred immune cell infiltration using an established immune inference method and evaluated the association between immune cell abundance and patient prognosis as well as common chromosomal abnormalities found in neuroblastoma. In addition, we evaluated co-infiltration patterns among distinct immune cell types.

**Results:**

The infiltration of naïve B cells, NK cells, and CD8+ T cells was associated with improved patient prognosis. Naïve B cells were the most consistent indicator of prognosis and associated with an active immune tumor microenvironment. Patients with high B cell infiltration showed high co-infiltration of other immune cell types and the enrichment of immune-related pathways. The presence of high B cell infiltration was associated with both recurrence-free and overall survival, even after adjusting for clinical variables.

**Conclusion:**

In this study, we have provided a comprehensive evaluation of immune cell infiltration in neuroblastoma using gene expression data. We propose an important role for B cells in the neuroblastoma tumor microenvironment and suggest that B cells can be used as a prognostic biomarker to predict recurrence-free and overall survival independently of currently utilized prognostic variables.

## INTRODUCTION

Neuroblastoma is the most common extracranial childhood cancer and accounts for 8%-10% of all childhood cancers^[1]^. It originates from neural crest progenitor cells and can consequently occur anywhere along the sympathetic nervous system with the most common location being the adrenal glands^[2,3]^. Neuroblastoma can develop sporadically or display autosomal dominant inheritance. The latter occurs most commonly due to familial mutations in the *ALK* or *PHOX*_*2*_*B* genes^[4,5]^. The prognosis of neuroblastoma patients has improved in recent years^[6]^. However, the 5-year survival rate of patients with high-risk disease is still below 50%^[2]^, highlighting the need for additional therapies.

Immunotherapy has recently led to a significant extension of survival rates in several adult cancers^[7]^. Although immunotherapy may also hold great promise for pediatric oncology, few clinical trials are currently being conducted in solid pediatric cancer types. The increased survival of patients with high-risk neuroblastoma following the success of anti-GD_2_ therapy exemplifies the potential of immunotherapy in neuroblastoma^[8]^. Thorough characterization of the tumor microenvironment (TME) is essential in identifying challenges and opportunities for additional immunotherapy use in neuroblastoma.

Due to the limited availability of fresh neuroblastoma tumor material and the practical challenges of in-depth immune analyses, several studies have recently investigated the composition of the immune cell infiltrate in neuroblastoma using gene expression datasets^[9–12]^. These studies have shown that several immune cell types infiltrate the neuroblastoma TME, including B cells, CD_8_+ T cells, NK cells, and macrophages^[9–12]^. In addition, these studies have also consistently shown that tumors displaying MYCN amplifications are significantly less immune infiltrated as compared to patients without MYCN amplifications^[9–12]^, which is consistent with more traditional immunohistochemistry (IHC) approaches^[9,10]^. However, several open questions remain. First, the majority of these studies only used a limited number of datasets, which potentially challenges external validity. Second, the association between individual immune cell types and patient prognosis has only been evaluated in a select number of studies for few immune cell types. Lastly, while the negative relationship between MYCN amplifications and immune infiltration is clear, the role of additional commonly altered chromosomal alterations in neuroblastoma and immune cell infiltration is unclear.

In this study, we aimed at providing a comprehensive investigation of immune cell infiltration in neuroblastoma using a large number of independent gene expression datasets. We evaluated the relationship between immune cell infiltration and patient prognosis, co-infiltration of immune cells, and the association between common chromosomal abnormalities and immune cell infiltration. We found a surprising role for B cell infiltration in both prognostic and co-infiltration analyses. In conclusion, our findings both confirm previous studies and propose an important role for B cells in the TME of neuroblastoma.

## METHODS

### Utilized data

A total of 11 publicly available gene expression datasets were utilized in this study. The Westerman^[13]^ and Oberthuer^[14]^ datasets were obtained from the European Molecular Biology Laboratory (EMBL) database under accession numbers E-TABM-_38_ and E-MTAB-_179_, respectively. The Henrich^[15]^, SEQC^[16]^, Kocak^[17]^, Wang^[18]^, Rajbhandari^[19]^, Lastowska^[20]^, and Ackerman^[21]^ datasets were obtained from the Gene Expression Omnibus (GEO) under accession numbers GSE_73517_, GSE_62564_, GSE_45547_, GSE_3960_, GSE_85047_, GSE_13136_, GSE_120572_, respectively. The Berwanger^[22]^ dataset was obtained from the PREdiction of Clinical Outcomes from Genomic Profiles portal (https://precog.stanford.edu/; accession: Berwanger_NB). The ICGC^[23]^ dataset was obtained through the ICGC portal (https://dcc.icgc.org/). Microarray datasets were provided as normalized expression at the probeset level in which some genes might be represented by multiple probesets. We converted probeset expression into gene expression values. Specifically, for one-channel arrays, we selected the probeset with the highest hybridization intensity across all samples to represent gene expression. For two-channel arrays, the average expression values of all probesets were calculated to represent gene expression. Datasets from one-channel arrays were further median normalized for each gene to transform intensities into relative expression values. Depending on availability, associated clinical data were obtained through EMBL, GEO, or the manuscript accompanying the dataset. See Supplementary Table 1 for detailed information and available clinical variables for each dataset.

### Immune cell inference

A detailed description of immune cell inference can be found in^[24,25]^. Briefly, patient-specific immune cell type inference was determined by evaluating the similarity between six predefined gene expression weight profiles (one for each immune cell type) and patient gene expression profiles using BASE^[26]^, a rank-based gene set enrichment method. High similarity between a patient’s gene expression profile and an immune cell weight profile resulted in high enrichment scores for that immune cell type for that particular patient. Due to the scale-free nature of resulting infiltration scores, immune cell infiltration scores are only comparable within each dataset and within an individual immune cell type.

### Survival analysis

Survival analyses were performed using the R survival package (version 3.1–8). Log-rank tests were performed to evaluate overall survival probabilities between two groups using the survdiff function. KaplanMeier (KM) plots were generated using the survfit function. Results from Cox proportional hazards (Coxph) models shown in KM plots were performed on continuous immune infiltration scores in a univariate regression model, using the coxph function from the survival package. Shown *P*-values were obtained from a two-sided Wald test. Forest plots were based on the results of multivariate Coxph models in which all variables specified in the figure panels were included and immune cell infiltration was dichotomized based on the median infiltration score.

### Statistical methods

The Spearman correlation coefficient (SCC) was reported for all correlation analyses as the assumptions underlying the Pearson correlation (i.e., normal distribution, homoscedasticity or linearity) were not met. SCC was calculated using the *R* function cor and significance was assessed using cor.-test. Immune cell infiltration variance explained by different chromosomal abnormalities was calculated using multivariate linear regression models using the lm and anova functions. The order of each of the four chromosomal abnormalities was randomly shuffled 100 times to obtain the standard deviation and mean variance. *P*-values smaller than 0.05 were considered significant. All analyses were conducted in *R* (version 3.6.2).

## RESULTS

### Immune infiltration in neuroblastoma is associated with patient prognosis

**Figure 1.**
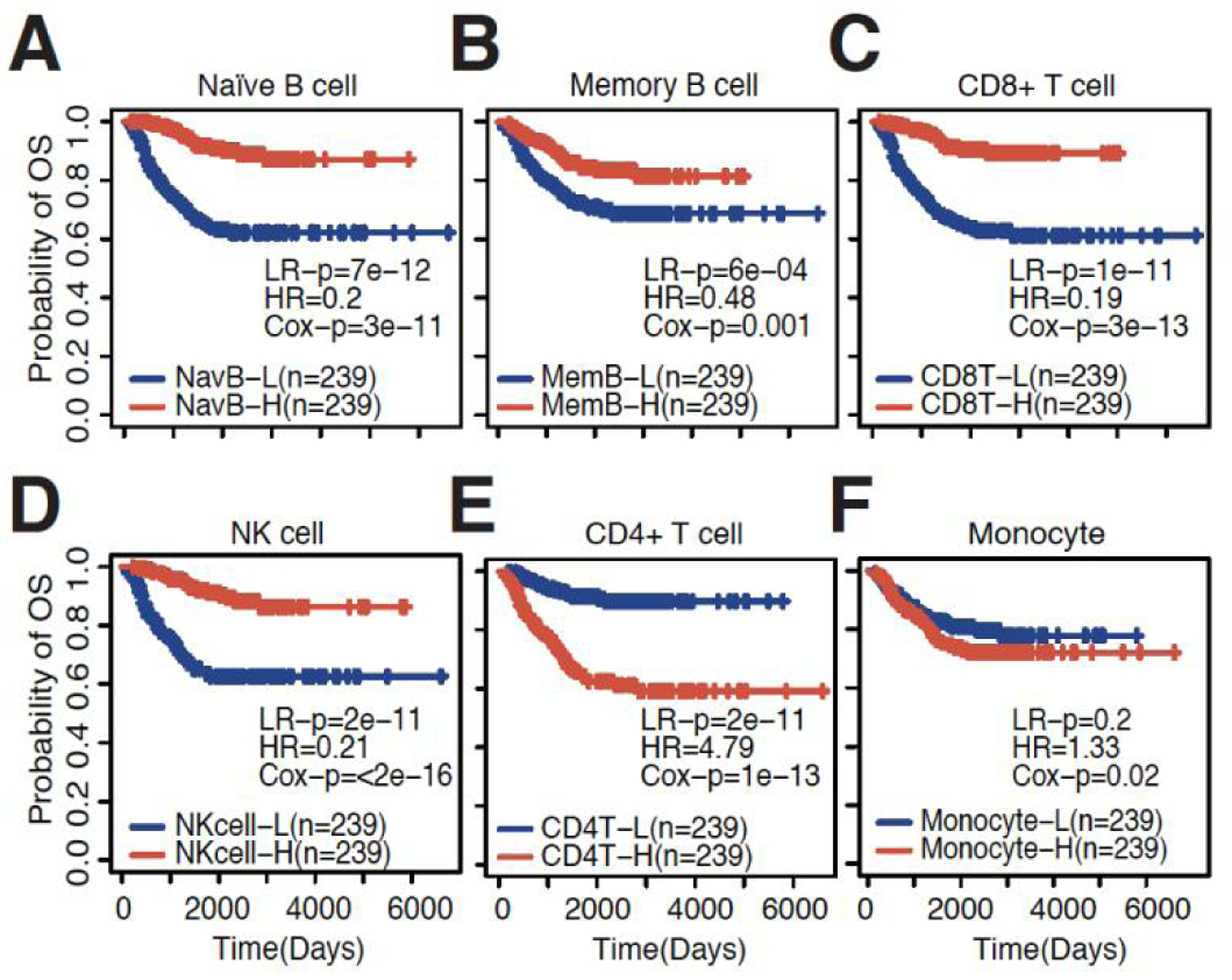
Immune cell inference is associated with patient prognosis. KM plots showing the association between high and low levels of (A) naïve B cells; (B) memory B cells; (C) CD8+ T cells; (D) NK cells; (E) CD4+ T cells; and (F) Monocytes in the Oberthuer dataset. LR-p: *P*-value calculated by Log-rank tests; HR: hazard ratio from univariate Coxph regression models; Cox-p: *P*-value calculated by univariate Coxph regression models.

To interrogate immune cell infiltration in neuroblastoma gene expression datasets, we inferred the abundance of six common immune cell types, naïve B cells, memory B cells, CD_4+_ T cells, CD_8+_ T cells, NK cells, and monocytes. This method has been well-established and validated in multiple studies^[24,25]^. We first evaluated immune cell infiltration in the Oberthuer neuroblastoma gene expression dataset. We observed that the infiltration of certain immune cells was positively associated with overall survival, while the infiltration of other immune cells was negatively associated. High abundance of naïve B cells, memory B cells, CD_8+_ T cells, and NK cells was significantly associated with longer overall survival [Figure 1A–D]. Conversely, high infiltration of CD_4+_ T cells was negatively associated with overall survival [Figure 1E]. The infiltration of monocytes was not significantly associated with survival based on a Log-rank test [Figure 1F]. Previous reports have suggested that few B cells infiltrate neuroblastoma tumors^[27,28]^ and little is known about their exact role in neuroblastoma. In addition, several studies have recently shown that B cells are crucial in mounting an effective anti-tumor immune response^[29–31]^. In our study, naïve B cells were highly significantly associated with survival as compared to the other major immune cell types, which sparked our interest.

**Figure 2.**
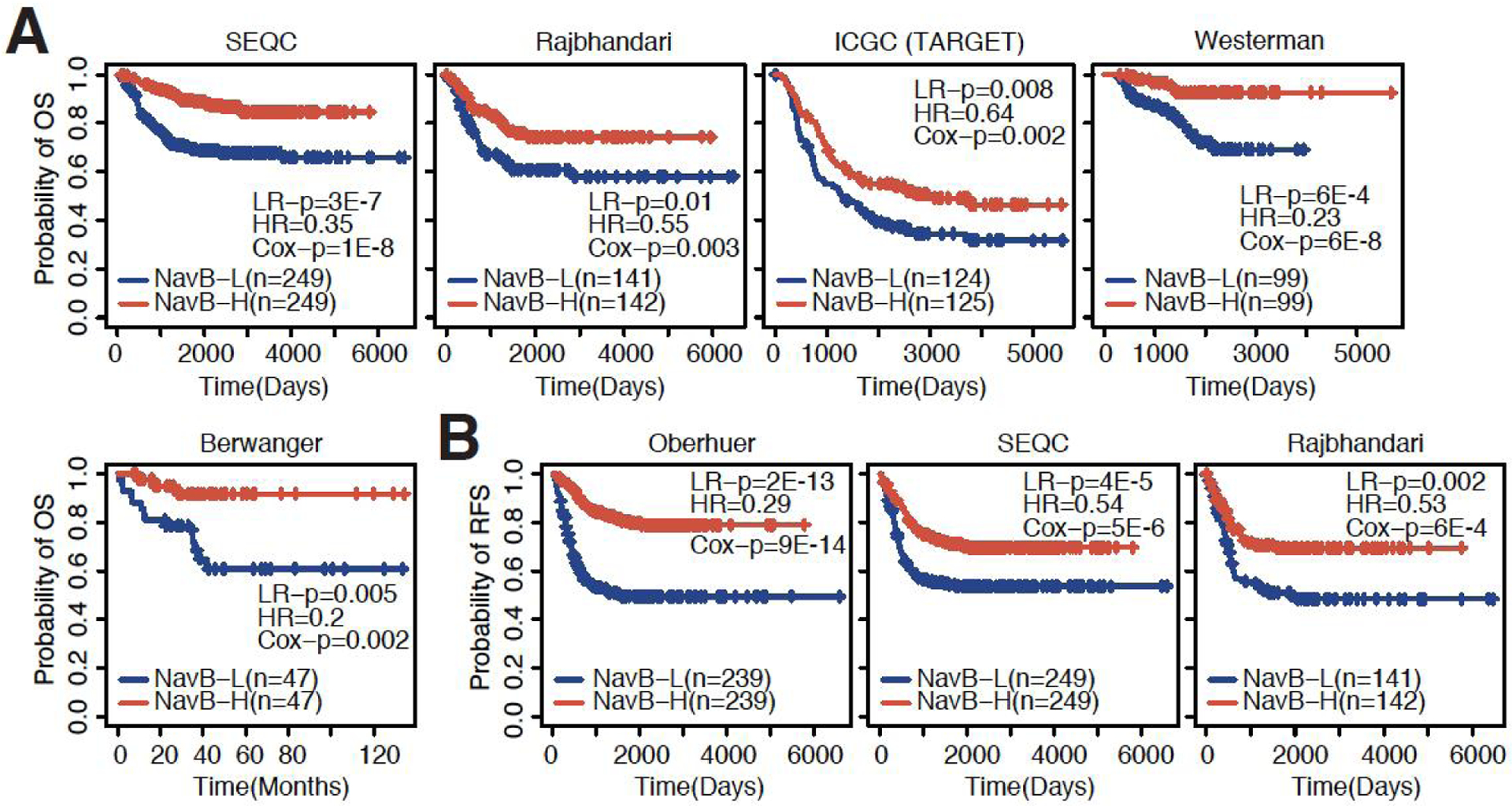
Naïve B cells consistently associated with overall and recurrence-free survival. (A) KM plots showing the association between high and low levels of naïve B cell infiltration and overall survival in the SEQC, Rajbhandari, ICGC (TARGET), Westerman and Berwanger datasets (datasets ordered based on sample size). (B) KM plots showing the association between high and low levels of naïve B cell infiltration and recurrence-free survival in the Oberthuer, SEQC, and Rajbhandari datasets (datasets ordered based on sample size). LR-p: *P*-value calculated by Log-rank tests; HR: hazard ratio from univariate Coxph regression models; Cox-p: *P*-value calculated by univariate Coxph regression models.

To validate our findings in the Oberthuer dataset, we collected several additional independent datasets containing patient survival information [Supplementary Table 1] and inferred immune cell infiltration for each dataset. We found a highly reproducible pattern where high infiltration of naïve B cells was consistently associated with better overall survival [Figure 2A]. Increased infiltration of CD_8+_ T cells and NK cells were also significantly associated with longer survival in more than half of the datasets [Supplementary Figure 1A]. In addition to overall survival, recurrence-free survival was also significantly longer in patients with high naïve B cell infiltration [Figure 2B]. In conclusion, these findings suggest that naïve B cells are a reliable prognostic indicator in neuroblastoma.

### Naïve B cells highly associated with survival independent of clinical variables

**Figure 3.**
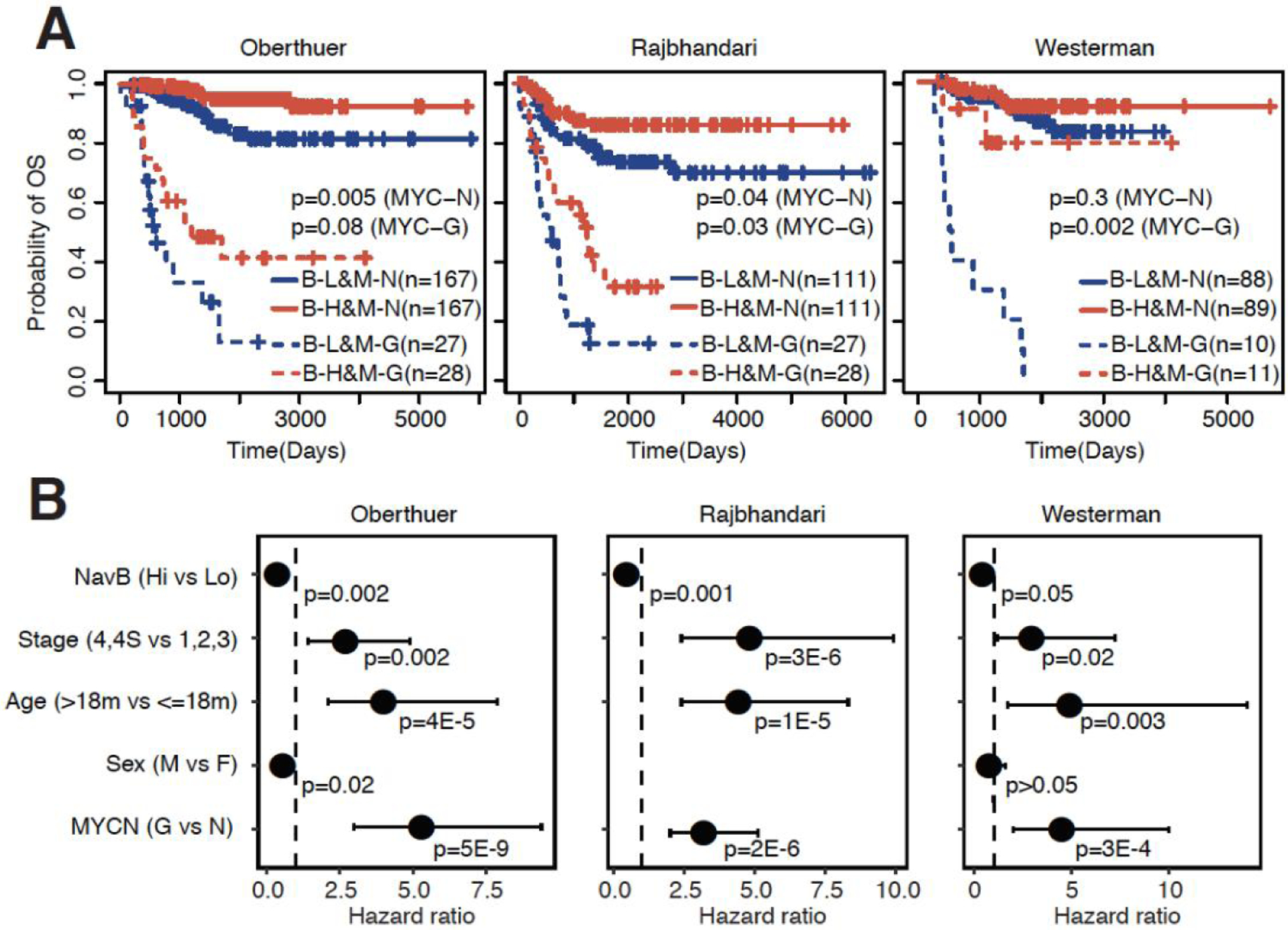
Naïve B cells associated with overall survival independent of clinical variables. (A) KM plots showing the association between high and low levels of naïve B cell infiltration and overall survival stratified based on MYC amplification status in the Oberthuer, Rajbhandari, and Westerman datasets (datasets ordered based on sample size). B-L: naïve B-low; B-H: naïve B-high; M-N: MYCN-Normal; M-G: MYCN-Gain; LR-p (MYC-N): *P*-value calculated by Log-rank tests comparing low and high naïve B infiltration within patients with MYCN-Normal status; LR-p (MYC-G): *P*-value calculated by Log-rank tests comparing low and high naïve B infiltration within patients with MYCN-Gain status. (B) Forest plots showing the association between naïve B cell infiltration and overall survival in the Oberthuer, Rajbhandari, and Westerman dataset using multivariate Coxph regression models adjusted for stage, age (> 18 months *vs*. ≤ 18 months), sex (male *vs*. female), and MYCN amplification status (MYC-Gain *vs*. MYC-Normal).

Naïve B cells were highly associated with patient prognosis in all evaluated datasets using univariate analyses. However, several clinical variables are also known to be highly associated with overall patient survival. The presence of MYCN amplifications (MYC-Gain) automatically classifies neuroblastoma as high-risk^[2,32]^ and patients with this amplification are treated more intensely to increase the probability of overall survival. We thus stratified patients based on MYCN amplification status and evaluated the association of naïve B cells with overall survival in MYC wild type and MYC-Gain patients [Figure 3A]. Patients exhibiting MYC-Gains indeed had much shorter overall survival, but patients with MYC-Gain and high naïve B cell infiltration did live significantly longer in two out of three evaluated datasets and the last datasets showed a trend of prolonged survival of patients with high naïve B cell infiltration [Figure 3A]. The infiltration of naïve B cells in patients who did not have MYC-Gains was also significant in two out of three datasets (*P* < 0.05) [Figure 3A].

In addition to MYCN amplification status, tumor stage and age are also important prognostic variables that are considered during risk stratification^[2,32]^. Even after adjusting for these prognostic clinical variables and MYCN amplification status, the infiltration of naïve B cells was still significantly associated with overall survival in all independent datasets [Figure 3B]. Irrespective of tumor stage and patient age, the infiltration of naïve B cells was consistently associated with longer patient survival. In addition, RFS was also significantly longer in patients with high naïve B cell infiltration, irrespective of MYCN-amplification status [Supplementary Figure 1B]. Adjustment of clinical variables and MYCN amplification status in multivariate Coxph models showed that high naïve B cell infiltration is significantly associated with prolonged RFS independent of adjusted variables [Supplementary Figure 1C]. In conclusion, the infiltration of naïve B cells is associated with prognosis in neuroblastoma irrespective of clinical variables and MYCN amplification status.

### The infiltration of naïve B cells in neuroblastoma is correlated with an immune hot tumor microenvironment

**Figure 4.**
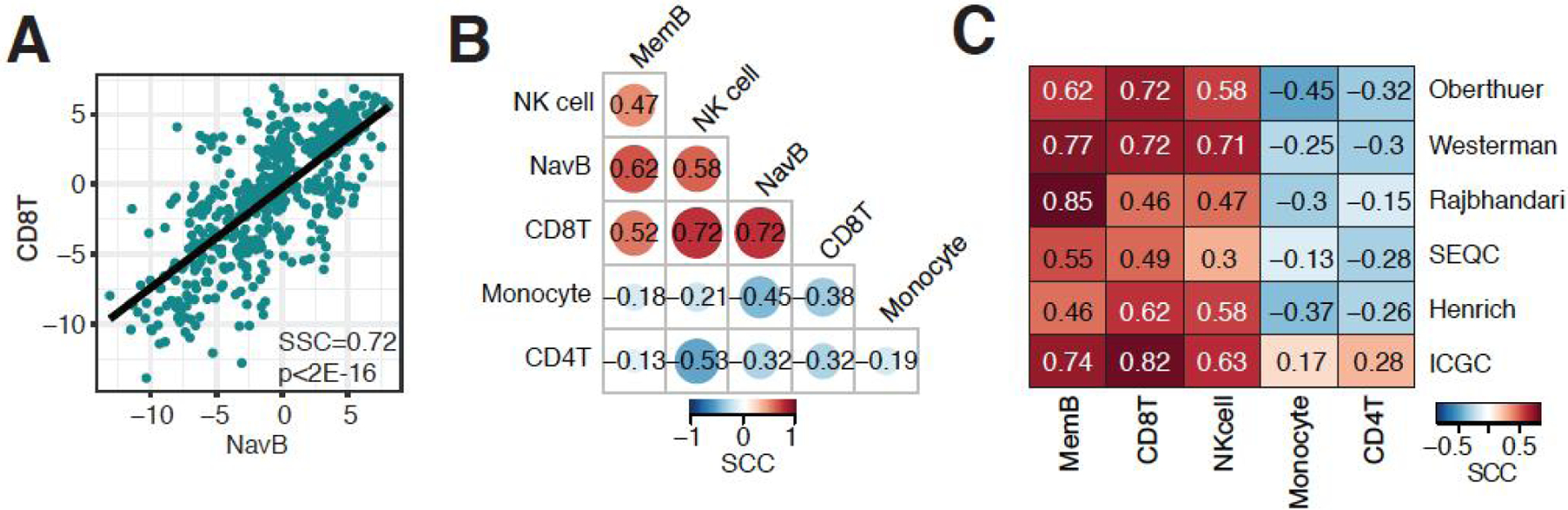
B cell infiltrated neuroblastoma associated with hot immune microenvironment. (A) Correlation between naïve B cell and CD8+ T cell infiltration in the Oberthuer dataset. (B) Correlation matrix of immune cell infiltration estimates in the Oberthuer dataset. (C) Correlation matrix of correlation coefficients between naïve B cell infiltration and 5 major immune cell types in 6 independent neuroblastoma datasets. SCC: Spearman correlation coefficient.

Previous studies have shown that different immune cell types are often present in a given tumor. Since naïve B cells were most consistently associated with prognosis, we evaluated if these cells are correlated with the presence of other immune cell types. CD_8+_ T cells are of major interest due to their essential role in an anti-tumor immune response^[33]^. We indeed found that naïve B cells are highly correlated with the presence of CD_8+_ T cells [Figure 4A]. In addition, we observed an interesting pattern in which naïve B cells were highly positively correlated with the presence of memory B cells, CD_8+_ T cells, and NK cells, but negatively correlated with monocytes and CD_4+_ T cells [Figure 4B]. The observed pattern in Figure 4B was highly reproducible in additional independent datasets [Figure 4C]. Five out of six datasets showed an identical pattern of high correlations with memory B cells, CD_8+_ T cells, and NK, but negative correlations with monocytes and CD_4+_ T cells. The last dataset showed positive correlations between naïve B cells and all other cell types, although the correlations with memory B cells, CD_8+_ T cells, and NK were much stronger as compared to the monocyte and CD4+ T cell correlations. As multiple CD_4+_ T cell subsets are recognized, we evaluated if we could further narrow down on the precise CD_4+_ T cell subset that is present in neuroblastoma. We utilized established CD4+ T cell subset marker genes^[34]^ and found that the inferred CD_4+_ T cells are most similar to activated CD_4+_ T cells. More specifically, both Th_1_ and Th_2_ signals were enriched [Supplementary Figure 1D]. In conclusion, it seems that the infiltration of B cells is associated with a hot TME in neuroblastoma.

### High naïve B cells infiltration associated with enrichment of immune-related pathways

**Figure 5.**
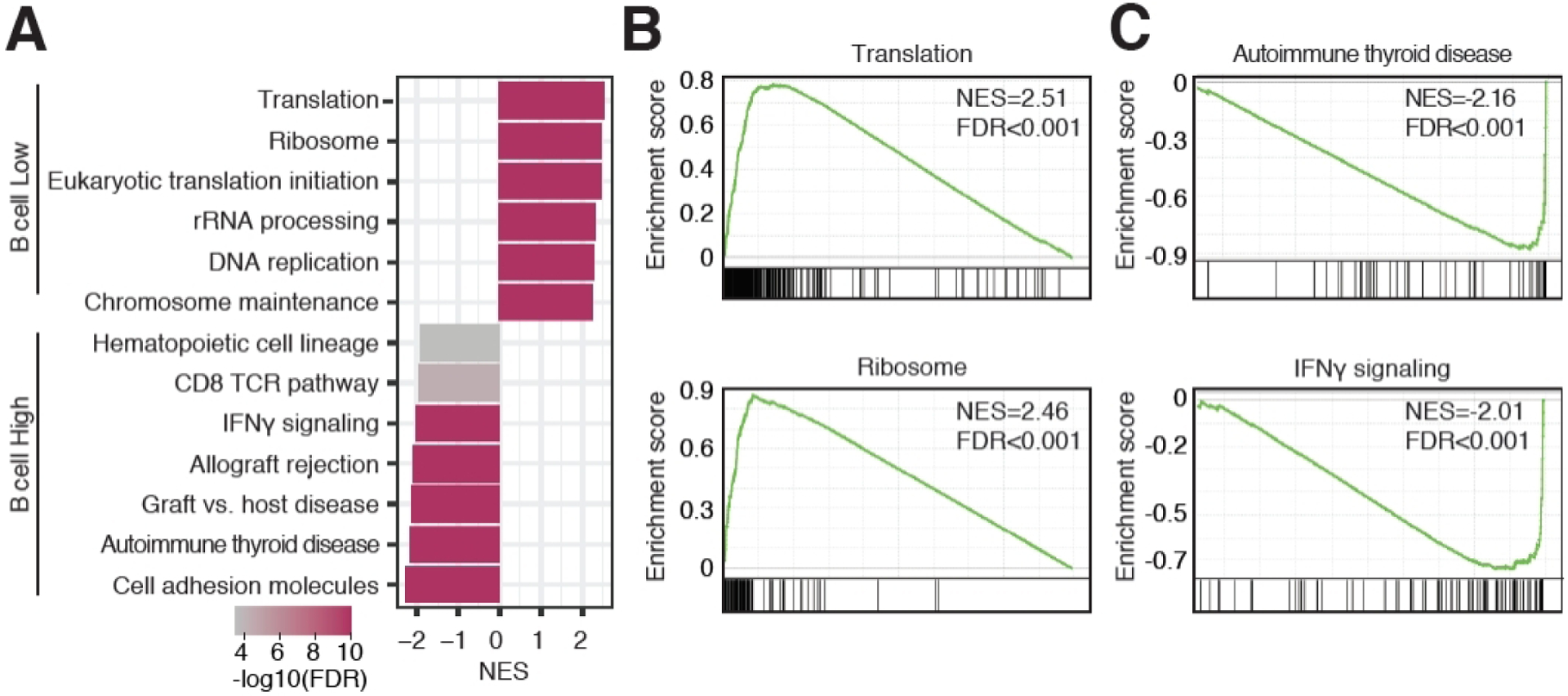
Immune-related pathways enriched in neuroblastoma tumors with high naïve B cell infiltration. (A) Bar plot showing normalized enrichment scores (NES) of pathways significantly enriched in tumors with low B cell infiltration (positive NES) and high B cell infiltration (negative NES). (B) Gene Set Enrichment Analysis plots of the Translation and Ribosome pathways. (C) Gene Set Enrichment Analysis plots of the Autoimmune thyroid disease and IFNγ signaling pathways.

To further investigate characteristics of the TME of B cell-infiltrated neuroblastoma, we separated patients based on low or high naïve B cell infiltration, using median naïve B cell infiltration as a separator. We performed Gene Set Enrichment Analysis to assess which pathways were enriched in either patient group. A distinct biological difference was observed, where pathways associated with cell proliferation were enriched in patients with low B cell infiltration, whereas immune-related pathways were enriched in patients with high B cell infiltration [Figure 5A]. For example, the Translation and Ribosome pathways were among the most highly enriched pathways in patients with low B cell infiltration [Figure 5B], potentially reflecting overall cell proliferation. Additional pathways related to cell proliferation, including eukaryotic translation initiation, rRNA processing, DNA replication, and chromosome maintenance were also among the most highly enriched pathways in patients with low B cell infiltration [Figure 5A]. Autoimmune thyroid disease and IFNγ signaling were among the most highly enriched pathways in patients with high B cell infiltration [Figure 5C]. Pathways related to transplant rejection were also enriched, including graft *vs*. host disease and allograft rejection [Figure 5A], likely reflecting the presence of an ongoing immune response in B cell-infiltrated tumors. In conclusion, low naïve B cell infiltration is associated with proliferative pathways whereas high naïve B cell infiltration is associated with immune-related pathways.

### Chromosomal abnormalities in relation to immune infiltration

**Figure 6.**
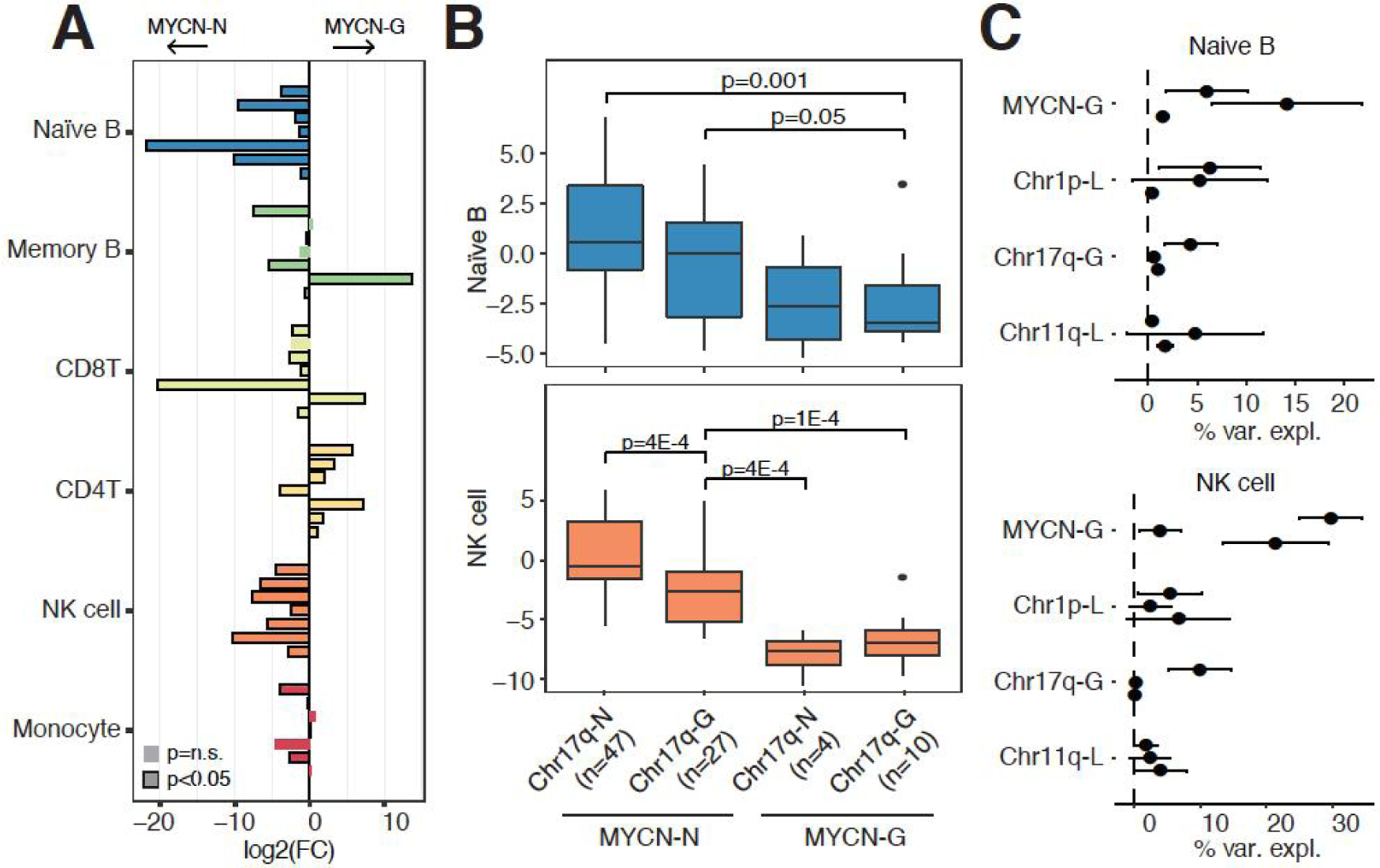
Immune cell infiltration and chromosomal abnormalities. (A) Bar graph showing the log2 (fold-change) [log2(FC)] comparing immune infiltration between tumors with normal MYCN copy number and tumors with MYCN amplifications. Each comparison was evaluated in seven independent datasets (top to bottom for each immune cell type): Westerman, Wang, SEQC, Rajbhandari, Henrich, Oberthuer, and Kocak. Significance calculated using Wilcoxon-rank sum tests. (B) Comparison of naïve B and NK cell infiltration in patients with or without Chr17q and MYCN chromosomal rearrangements in the Wang dataset. Significance calculated using Wilcoxon-rank sum tests. (C) Percentage of immune cell variance explained by 4 chromosomal rearrangements. Horizontal bars indicate the standard deviation and solid dots indicate the mean variance explained of 100 iterations in which the order of variables was randomly shuffled in each iteration. Each comparison was evaluated in 3 independent datasets (top to bottom for each chromosomal rearrangement): Wang, Lastowska, and Henrich.

Previous studies have investigated the relationship between specific copy number variations and immune cell infiltration in neuroblastoma. Several studies have suggested that neuroblastoma tumors with MYCN amplifications are poorly infiltrated^[9–12]^. We indeed confirmed the negative relationship between MYCN amplifications and immune cell infiltration in seven independent datasets [Figure 6A]. Naïve B cells and NK were the most consistent cell types associated with MYCN amplification status, showing significantly lower immune infiltration in MYCN amplified samples in all seven datasets. In addition to MYCN amplifications, other chromosomal abnormalities commonly occur in neuroblastoma. Only a small number of datasets contained information on Chr1p, Chr11q, and Chr17q status, the most commonly altered chromosomal abnormalities in neuroblastoma^[2,32]^. Since MYCN amplification is strongly associated with immune infiltration, we separated samples based on MYCN status and each of the other chromosomal rearrangements. Although we did observe some differences between naïve B and NK cell infiltration in samples with and without Chr17q gains, MYCN amplification status was much more significantly associated with the infiltration of these immune cells [Figure 6B]. TERT rearrangements and ATRX mutations are also commonly observed in neuroblastoma^[2,32]^. While no difference in immune cell infiltration based on TERT rearrangement status was observed, patients with ATRX mutations had significantly lower levels of CD_4+_ T cell and higher levels of monocyte infiltration as compared to patients without ATRX mutations [Supplementary Figure 2A–B].

In addition to assessing specific genotypic groups, we also assessed how much immune cell variation can be explained by individual chromosomal rearrangements. Since the order of variables affects the percentage of variation explained by each variable, we randomly shuffled the order of variables 100 times and calculated the mean and standard deviation of the percentage of immune cell variance explained (see Methods). There was considerable variation between datasets, but MYCN amplification status again showed the most consistent results, especially in naïve B cell and NK cell infiltration [Figure 6C]. MYCN amplification status explained approximately 10% of naïve B cell infiltration when considering four chromosomal rearrangements in the model, while MYCN status accounted for approximately 25% of NK cell infiltration in two out of three datasets [Figure 6C].

## DISCUSSION

The presence of tumor infiltrating leukocytes is indicative of a host immune response to tumors and infiltrating immune cells have been shown to be predictive of clinical outcomes for neuroblastoma patients^[9,35]^. In our study, we show that several immune cell types are associated with recurrence-fee survival (RFS) and overall survival, most notably naïve B cells, NK cells, and CD_8+_ T cells. We have expanded on previous immune inference studies by utilizing a large number of gene expression datasets, as well as by evaluating the association between prognosis and several immune cell types. We propose a previously unappreciated role for naïve B cell abundance in neuroblastoma, which is highly associated with survival and a hot TME.

Previous studies have suggested that only a small number of B cells infiltrate in neuroblastoma tumors^[27,28]^. However, larger numbers of B cells might reside just outside the tumor. The presence of organized lymphoid structures and B cell follicles at the edges of neuroblastoma tumors have been observed^[27]^. We hypothesize that the small number of tumor infiltrating B cells might originate from these B cell-enriched locations that might not always be captured during biopsies or tissue sections. This hypothesis is in line with recent observations of B cells in other cancer types, where B cell follicles can reside at the tumor margin^[29–31]^. The presence of these B cell structures is highly associated with survival and an effective anti-tumor immune response^[29–31]^.

A number of B cell-related mechanisms are operational in the TME. Antigen presentation by B cells^[36]^ is likely a major contributor to the reported positive association between B cell infiltration and patient survival. For example, the occurrence of antigen-specific interactions between T cells and B cells in tumor B cell structures promote CD_8+_ T cell cytotoxicity in the TME^[37,38]^. Another anti-tumor mechanism is the secretion of tumor-specific antibodies that mediate opsonization, antibody-dependent cellular cytotoxicity by NK cells, or promote tumor cell phagocytosis by macrophages and granulocytes^[39]^. Lastly, the secretion of cytokines, including IFNγ and IL-12, by B cells promotes further activation of anti-tumor CD_8+_ T cells and NK cells^[39]^.

We confirmed the findings of previous studies which showed that MYCN amplified neuroblastoma tumors have significantly lower immune cell infiltration compared to patients without MYCN amplifications^[9–12]^. When separating patients without and with MYCN amplifications, we still observed that naïve B cell infiltration was associated with overall survival and RFS. This is consistent with a previous study that reported that certain immune characteristics are associated with patient survival irrespective of MYCN amplification status^[40]^. B cell infiltration could thus be used as a prognostic marker in neuroblastoma in addition to commonly utilized prognostic indications such as age, stage and MYCN amplification status. Consistently, we observed significant associations between B cell infiltration and prognosis when adjusting for clinical variables and MYCN status.

Although our study provides important insights into immune infiltration in neuroblastoma, a few limitations should be noted. First, all of our findings are based on gene expression data, which might not always recapitulate protein expression. Protein-based approaches such as immunohistochemistry should corroborate our findings of high B cell infiltration in patients with a better prognosis. Evaluation of the presence of tertiary lymphoid structures adjacent to neuroblastoma tumors should be performed as well. Second, although we attempted to evaluate the association between immune cell infiltration and common chromosomal abnormalities in neuroblastoma, notably Chr1p deletion, Chr11q deletion, and Chr17q gain, only few studies contained this information. Additional studies with available information on chromosomal deletions and gains should be performed to validate our findings. Lastly, our prognostic analyses were all performed retrospectively and prospective studies should evaluate the exact value of B cell as a prognostic biomarker in neuroblastoma.

In conclusion, we have provided a comprehensive evaluation of immune cell infiltration in neuroblastoma using gene expression data. The infiltration of naïve B cells, NK cells, and CD_8+_ T cells is associated with better prognosis in neuroblastoma among which naïve B cells are the most consistent indicator of prognosis. Based on further analyses, we propose a critical role for B cells in the neuroblastoma TME. The presence of high B cell infiltration is associated with an immune-infiltrated TME and could be used as a prognostic biomarker to predict recurrence-free and overall survival independently of currently utilized prognostic variables.

## Supplementary Material

Supplementary Meterial
