# Supplementary material for: B cell infiltration is highly associated with prognosis and an immune-infiltrated tumor microenvironment in neuroblastoma
Source: J Cancer Metastasis Treat. Author manuscript; Available in PMC 2021 Aug 26. (PMC8389852; doi:10.20517/2394-4722.2021.72)
Supplement: Supplementary Meterial [file NIHMS1719673-supplement-Supplementary_Meterial.pdf]

Supplementary Figures

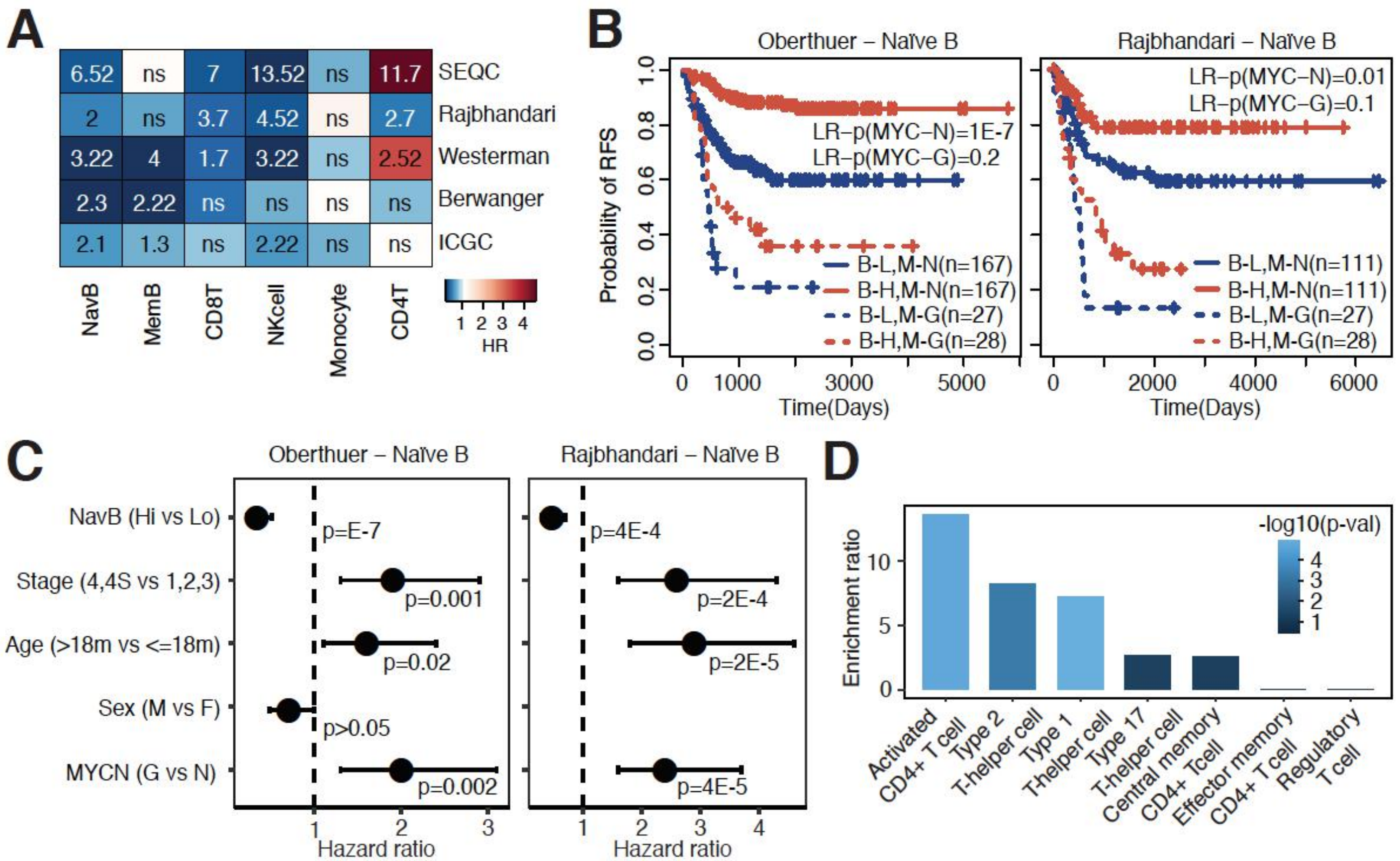

Suppl. Figure 1. Association between immune cell infiltration levels and prognosis in neuroblastoma. A. Heatmap depicting -log10(p-values) of log-rank tests for 5 datasets and 6 immune cell types. Color indicates magnitude of hazard ratios. **B.** KM plots showing the association between high and low levels of naïve B cell infiltration and recurrence-free survival stratified based on MYC amplification status in the Oberhuer and Rajbhandari datasets. B-L = naïve B-low, B-H = naïve B-high, M-N = MYCN-Normal, M-G = MYCN-Gain. LR-p(MYC-N) = p-value calculated by Log-rank tests comparing low and high naïve B infiltration within patients with MYCN-normal status. LR-p(MYC-G) = p-value calculated by Log-rank tests comparing low and high naïve B infiltration within patients with MYCN-Gain status. **C.** Forest plots showing the association between naïve B cell infiltration and recurrence-free survival in the Oberthuer and Rajbhandari datasets using multivariate Coxph regression models adjusted for stage, age (>18 months vs. <=18 months), sex (male vs. female), and MYCN amplification status (MYC-Gain vs. MYC-Normal). **D.** Enrichment of CD4+ T cell subset marker genes in the inferred CD4+ T cell signature. P-value calculated using hypergeometric tests. CD4+ T cell marker genes obtained from [34].

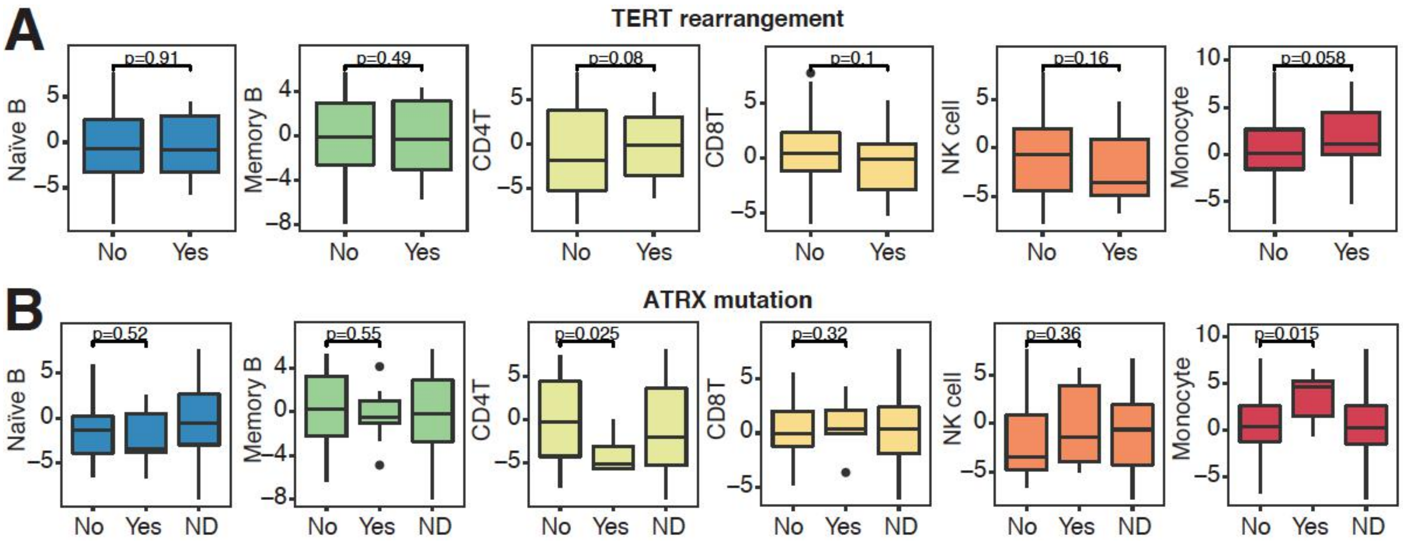

Suppl. Figure 2. Association between TERT rearrangements or ATRX mutations and immune cell infiltration in neuroblastoma. A. Comparison of immune cell infiltration in patients with or without TERT rearrangements in the Ackermann dataset. **B.** Comparison of immune cell infiltration in patients with or without ATRX mutations in the Ackermann dataset. Significance calculated using Wilcoxon rank-sum tests. ND = no data.

Supplementary Tables

Supplementary Table 1. Overview of datasets utilized in this study

First Author/ Consortium	PMID	GEO	EMBL	N	Matching clinical data	Overall survival	Recurrence free survival	Age	Stage	Grade	Sex	NMYC copy	Chr 1p	Chr 11q	Chr 17q	TERT	ATRX	Platform
Berwanger	12450793	N/A	N/A	94	94	Yes	No	No	No	No	No	No	No	No	No	No	No	Microarray
Westerman	18851746	N/A	E-TABM-38	251	198	Yes	Yes	Yes	Yes	No	Yes	Yes	No	No	No	No	No	Microarray
Henrich	27635046	GSE73517	N/A	105	105	No	No	Yes (<1.5y, >1.5y)	Yes	No	No	Yes	Yes	Yes	Yes	No	No	Microarray
Oberthuer	20676065	N/A	E-MTAB-179	478	478	Yes	Yes	Yes	Yes	No	Yes	Yes	Yes	No	No	No	No	Microarray
ICGC (TARGET)	23334666	N/A	N/A	249	249	Yes	No	Yes	Yes	Yes	Yes	No	No	No	No	No	No	RNA-seq
SEQC	25254650	GSE62564	N/A	498	498	Yes	Yes	Yes	No	No	No	No	No	No	No	No	No	RNA-seq
Kocak	24737690	GSE45547	N/A	649	649	No	No	Yes (<18m, >18m)	Yes	No	Yes	Yes	No	No	No	No	No	Microarray
Wang	16778177	GSE3960	N/A	102	102	No	No	Yes	Yes	No	No	Yes	Yes	Yes	Yes	No	No	Microarray
Rajbhandari	29510988	GSE85047	N/A	283	283	Yes	Yes	Yes	Yes	No	No	Yes	No	No	No	No	No	Microarray
Lastowska	17533364	GSE13136	N/A	30	30	No	No	Yes	Yes	No	Yes	Yes	Yes	Yes	Yes	No	No	Microarray
Ackermann	30523111	GSE120572	N/A	394	394	No	No	Yes	Yes	No	No	Yes	No	No	No	Yes	Yes	Microarray
